# Delusional misidentification syndrome and criminal acting out: A case report of maternal filicide

**DOI:** 10.1002/ccr3.4425

**Published:** 2021-07-12

**Authors:** Hanen Ben Ammar, Ghada Hamdi, Lina Brahmi, Yomn Naceur, Emira Khelifa, Rania Felhi, Leila Mnif

**Affiliations:** ^1^ Faculty of Medicine of Tunis El Manar University Tunis Tunisia; ^2^ Razi Hospital Manouba Tunisia

**Keywords:** Capgras syndrome, delusional misidentification, filicide, infanticide

## Abstract

Delusional misidentification syndromes are associated with danger and may lead to homicide. Early assessment of the danger is a key part of the psychiatric examination. Compliance to treatment in psychosis is important to avoid the devastating consequences, for the perpetrator, the victim, and the community.

## Graphical abstract

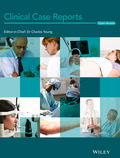

## 1 INTRODUCTION

We report the case of a woman with schizoaffective disorder, killing her child under the effect of an impulse motivated by Capgras syndrome in a strange crime scene including evisceration and enucleation. This case aims to better understand the association between misidentification syndromes and homicide and to promote preventive measures.

The issue of dangerous crimes has attracted the interest of researchers in different fields. Several researchers suggest the existence of a link between violence, homicide, and mental illness.^1^, ^2^, ^3^ This association is more observable in relation to severe mental disorders, such as psychotic ones. Delusions and hallucinations are among factors motivating crimes.^4^, ^5^ Delusional misidentification syndromes (DMS) are defined as a group of disorders involving a belief that the identity of a person, object, or place has been replaced or altered. These psychopathological phenomena, relatively misdiagnosed, can be observed in psychiatric and neurological illnesses.^6^ They include Capgras syndrome, Frégoli syndrome, subjective doubles syndrome, and intermetamorphosis delirium. Besides, they can be associated with patient violent behavior.^7^ Some intrafamily crimes, in particular murder of a misidentified familiar person (such as parricide, matricide, fratricide), have been described as associated with these syndromes.^8^, ^9^

Here, we present the case of a mother killing her child. This crime was motivated by a morbid impulse due to DMS, in a strange crime scene including evisceration and enucleation. Through this case, we seek to better understand the role of the DMS in criminal acting out and to highlight the importance of violence risk assessment in mentally disordered patients.

## 2 CASE REPORT

A 35‐year‐old woman was hospitalized for observation as part of a forensic psychiatric assessment, according to the 25 bis Article of the 2004/40 Tunisian mental health and hospital conditions’ law. This hospitalization aimed to evaluate her criminal responsibility for the murder of her daughter.

The patient is the younger of two siblings. She grew up in a family with a high socioeconomic level living in a rural community. Her mother died at the age of 64 years, six months prior to the current episode. She graduated in Industrial Economics at the age of 28, and she has remained unemployed since. She is married and a mother of three, aged 9 years, 4 years, and 18 months.

Concerning her premorbid functioning, the patient was dynamic, curious, independent, sociable, and well‐suited. A quiet and balanced atmosphere reigned within her family. She described a conflictual relationship with her mother, without the latter being repulsive. She spoke coldly of her mother, who had just died, describing her as ambivalent; she was both protective and unfair since she always preferred the patient's brother. Her father was described as rigorous, meticulous, and anxious. She reported good marital harmony and a warm relationship with her children.

The interview with the family revealed a behavioral change since the “Tunisian revolution” in 2011, characterized by social withdrawal, emotional coldness, exaggerated interest in politics, and extremism in religious practice. All of it was tolerated and even trivialized by her husband. Five years later, the patient was seen by a psychiatrist who prescribed risperidone at a dose of 4 mg per day. The treatment was refused by the patient. Then, she was admitted under the request of her family in a psychiatric hospital, for a fruitful delusional episode characterized by themes of persecution and influence, with hallucinatory and interpretative mechanisms. This delusional episode was triggered by psycho‐emotional stress: arguing with her eldest daughter's teacher, forcible confinement for nearly an hour in the school with her two children, followed by an aggressive arrest at the police station. The diagnosis of schizoaffective disorder was held requiring antipsychotics and mood stabilizers. After being discharged, the follow‐up and monitoring were discontinued, and she did not consult again until the murder. The entourage reported a change in the patient's behavior after the death of her mother. She became fulfilled and jovial.

On the day of the murder, the patient was at her father's home with her two daughters. In the late afternoon, she suddenly got agitated, and she banged her youngest daughter violently against the wall several times. Then, she stabbed her with two different knives.

The examination and autopsy of the infant revealed:

- Multiple bruises on the inside of the scalp, associated with subarachnoid hemorrhage and subdural hematoma, whose appearance is consistent with injuries caused by a blunt object that had hit the head.
- Multiple transfixing wounds of both eyeballs (the reflex zones) caused by a pungent and sharp object (emergence of several wounds with persistence inter‐membranous flanges at both eyelids).
- A left pneumothorax compression in connection with penetrating wounds in the left chest.
- Multiple deep ecchymoses in the epigastric region of the abdomen, back, and lumbar region, whose appearance is consistent with damage caused by a pungent and sharp object.
- Postmortem wounds in the abdominal organs (liver, right kidney, stomach, and intestine). A bite‐like wound was found on the liver.

The infant's death is related to head trauma and chest wounds.

On the first day of hospitalization, the patient was stammering, asthenic and would only remember the smell of blood. She also asked whether the deceased girl was indeed her own daughter. Throughout the interviews, her anxiety was intense when she talked about her act. She accused the police of having substituted her daughter to be able to monitor her. She saw the deceased daughter as a double of her daughter, physically identical, but it was a dangerous object that threatened her family. A destructive fury then fell on this danger.

She confessed that during her pregnancy, she considered her daughter as “blessed.” She wanted to give birth at home out of fear of substituting her child with another, something that was not allowed by her family. Since the birth of her child, she has had doubts about her identity. At the same time, she said “this girl is my spitting image.”

The patient's physical examination, biological tests, electroencephalogram, and cerebral magnetic resonance imaging were free of abnormalities.

The presence of a mental disorder causing a lack of judgment led to the conclusion of criminal irresponsibility objectified by the forensic psychiatry expert.

## 3 DISCUSSION

The pathological nature of filicide, in this observation, appears through the psychiatric history of the patient and the murderous act committed. Children murdered by their parents are usually grouped under the term “infanticide.” Several classifications have been proposed, including that of JP Resnick who distinguishes neonaticide (the killing of the infant within the first 24 hours following birth) from filicide (the murder of a child older than 24 hours by a parent).^10^

J. Delay et al established a clinical classification of pathological infanticide: melancholy infanticide, infanticide during the period of puerperium, accidental infanticide, and infanticide during delusional experiences such as the case of our patient.^11^ Delusions of this patient were chronic, insidious, and had a mystical aspect. Thus, they were credible for her husband. Furthermore, the weird and impulsive nature of this homicide reveals major anxiety. The data and findings of the autopsy of the victim justified the psychotic nature of the patient's disease. The amnesia of the facts recorded in the register of a confusing episode might be misdiagnosed with a seizure. This remains improbable because of the normal neurological explorations.

The category named “pathological filicide” is well described in the literature, where violence aims to control the perceived threat, such as our patient, whose deeds place her in a space between the monster and the sick.^12^ This is a dive into the clinic of horror. This murder seems absurd, illogical, outrageous, out of proportion with avowed causes, or even without apparent motive. It is far from the usual criminal motivations. It would refer to a pathological dimension of the act, such as unmotivated brutal, hermetic, and cold murders. This woman with chronic psychosis, facing the challenge of controlling the unthinkable, worries, and illusions related to the imminent catastrophic danger, was unable to control her impulses. She committed her crime without premeditation, under the effect of a morbid impulse supported by a delirious illusion of look‐alikes that corresponds to a Capgras syndrome. Indeed, she captured the likeness of the body to her daughter, but she disregarded her identity. She was convinced that her daughter has been replaced by a look‐alike that returned a sense of significant persecution and fear of annihilation by bad objects. Thus, to preserve and protect herself, she resorted to physical violence because of profound failures of mentalizing.

Marinopouls S supports that the child's death results from the mother's deficiency dimension of psycho‐affective life causing a denial of the emotional life and a denial of the child. In fact, our patient doubted the authenticity of her descendant from birth, accusing the police of substituting her child.^13^ On the other hand, the patient said that her child was her spitting image. In this sense, Wieder reports a situation where “the violent act had not targeted her child, but the bad parts of self”.^14^

In other cases, filicide could be considered a suicidal equivalent, especially among mothers who projected their psychotic symptoms into their children. According to Maria‐Valeria Karakasi et al who presented the case of prepartum psychosis which escalated to the endpoint of neonaticide, psychotic mothers tend to project their unacceptable psychiatric symptoms into their children and may murder them in order to “kill” their own symptoms.^15^

The child, then considered a duplicate of herself, is included in a suicidal urge, finally becoming the only victim.^16^

Through this observation, it is important to note that the central role of delusions in the genesis of crime; first in imaginative reconstructions about her mother, accentuated by prejudiced thoughts, and later about her daughter, who was thought to be substituted. The filicide crime is related to a defect in the filiation link between the mother and her child as if there was a confusion of generations, between a desire of matricide and a desire of infanticide. The issue of confusion of generations, mother and daughter, arises in the case of our patient. In fact, she killed her daughter in the same year of the death of her mother. This confusion might sign a psychotic break.

C. Ley et al reported that homicidal acts break definitively the chain of descent already weakened in the psyche of the murderer.^17^ Simonnot et AL confirmed that the occurrence of infanticide can be understood only by involving psychodynamic lighting in the context of serious disorders affecting the functioning of an entire family.^18^ According to some authors, women's violence reflects the presence of a destructive mother, and these committing filicides have a defective relationship with their mothers.^19^, ^20^ Thus, through the internalization of the destructive maternal picture, acting out filicide would form a dynamic of intergenerational repetition.

The peculiarity of our case is the fact that the act of filicide was supported by a DMS precisely Capgras syndrome. Although Capgras syndrome was described as a specific risk factor for homicidal behavior against the misidentified person,^7^, ^8^ to our knowledge, very few similar cases have been reported in the literature, and limited data have explored the association between DMS and infanticide.^21^, ^22^, ^23^

Capgras syndrome, or look‐alikes illusion, corresponds to the belief that one or more people close to the patient in the matter have been replaced by doubles.^7^ These doubles are physically identical to the originals but are psychologically different. The latter are described as being hostile, malicious, and persecutory.^7^ Intrafamily homicides were often described in cases of Capgras syndrome.^24^ The perpetrators are often male, and it is mostly a well‐planned act contrary to our case, where the abusive fierceness to the victim could be explained by the strange nature of the delusional ideation, the derealization at the crime time, and a bizarre urge to silence the victim. A recent study that collates all cases of Capgras' delusion since the first case report was published in 1963, supports the fact that derealization has been described as an occasional component in the genesis of the delusional belief.^23^ It is precisely the combination of Capgras delirium and the persecution delirium against the cops, which was at the origin of this imminent danger. The prognosis did not depend only on her disease, but a whole environment and a whole bundle of situational factors, namely: the important diagnostic and therapeutic delay due to the uneasily detected signs of her disease, and the trivialization from her family side though they were of a good social level; in addition to non‐adherence to treatments.

Therefore, clinical implications ensue from this case and which consist in the importance of assessment of violence associated with mental illness, and the interest in developing tools for psychological exploration and evolution's monitoring of the psychotic diseases and adapting treatments. In this sense, some authors highlight the importance of assessment of violence associated with DMS, and the interest in developing more in‐depth studies in order to better understand the mechanisms underlying these disorders and the plausible association between these syndromes and overkill homicide, especially since the data in the literature on the subject are still limited, to finally allow adapted therapeutic perspectives.^21^, ^25^, ^26^, ^27^

## 4 CONCLUSION

DMS is a psychopathological phenomenon with an increasing risk of intrafamily crimes such as filicide. Acute symptomatology impairment of reality control and delusions may increase this risk. The identification of the symptoms is crucial to maintain both maternal and infant health. An in‐depth psycho‐criminological study could provide important information on the nature of the disease, especially on the social errors committed, as well as detect clues to protect children.

## CONFLICT OF INTEREST

All authors declare that they have no conflicts of interest to disclose.

## AUTHOR CONTRIBUTIONS

HBA and GH: conceived the ideas and led the writing. LB, YN, RF, and EK: involved in writing. LM: did the editing.

## Data Availability

Authors declare that this paper does not include supplementary data.
